# Functional shifts in estuarine zooplankton in response to climate variability

**DOI:** 10.1002/ece3.6793

**Published:** 2020-09-29

**Authors:** Anna Jansson, Riina Klais‐Peets, Evelina Grinienė, Gunta Rubene, Anna Semenova, Aleksandra Lewandowska, Jonna Engström‐Öst

**Affiliations:** ^1^ Novia University of Applied Sciences Ekenäs Finland; ^2^ EcoStat Ltd Tartu Estonia; ^3^ Marine Research Institute Klaipėda University Klaipėda Lithuania; ^4^ Fish Resources Research Department Institute of Food Safety, Animal Health and Environment Riga Latvia; ^5^ Atlantic Branch of ‘Russian Federal Research Institute of Fisheries and Oceanography’ (AtlantNIRO) Kaliningrad Russia; ^6^ Tvärminne Zoological Station University of Helsinki Hangö Finland

**Keywords:** climate change, complexity, functional group richness, functional traits, oxygen, salinity, zooplankton community

## Abstract

1. Functional traits are becoming more common in the analysis of marine zooplankton community dynamics associated with environmental change. We used zooplankton groups with common functional properties to assess long‐term trends in the zooplankton caused by certain environmental conditions in a highly eutrophicated gulf.
2. Time series of zooplankton traits have been collected since the 1960s in the Gulf of Riga, Baltic Sea, and were analyzed using a combination of multivariate methods (principal coordinate analysis) and generalized additive models.
3. One of the most significant changes was the considerable increase in the amount of the zooplankton functional groups (FGR) in coastal springtime communities, and dominance shifts from more complex to simpler organism groups—cladocerans and rotifers.
4. The results also show that functional trait organism complexity (body size) decreased considerably due to cladoceran and rotifer increase following elevated water temperature. Salinity and oxygen had negligible effects on the zooplankton community.

## Graphical abstract

Time series of zooplankton traits were collected since the 1960s in the Gulf of Riga, Baltic Sea, and were analysed using general additive model, principal component analysis, and multivariate model. One of the most significant changes was the considerable increase in the amount of the zooplankton functional groups (FGR) in coastal springtime communities, and dominance shifts from more complex to simpler organism groups—cladocerans and rotifers. The results also show that the functional trait organism complexity (body size) decreased considerably due to cladoceran and rotifer increase following elevated water temperature.

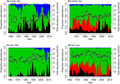

## 1 INTRODUCTION

To detect changes in multispecies communities caused by environmental changes, the communities have traditionally been studied based on taxonomic classification of species, focusing on their abundance and diversity (Sherman, Solow, Jossi, & Kane, 1998). This approach, however, does not provide immediate information on the functioning and the ecological properties of the communities, but only on their composition and relative abundance. Functional traits can be used to provide information on how changes in the community might lead to changes in the functioning of the system (Hébert, Beisner, & Maranger, 2017).

The changes in environmental conditions modify populations and communities by impacting species distribution ranges, abundance and dominance relations, food‐web structures, and behavioral and physiological functions of species. Through these effects, the structure and functioning of whole ecosystems can be altered (Doney et al., 2012; Thomas et al., 2004). In the Baltic Sea area, like elsewhere in the world, warming and increasing precipitation are both accelerating due to global climate change. Warming of the Baltic Sea has continued since 1,860 (Belkin, 2009; MacKenzie & Schiedek, 2007) with a rate of 1°C per decade in all subbasins (BACC II Author Team, 2015; Lehmann, Getzlaff, & Harlass, 2011). A salinity decrease of ca. 1–2 units is expected by the end of this century mainly due to more frequent rainfall and the subsequent rise in freshwater runoff from the catchment area (Holopainen et al., 2016; Meier et al., 2012). The increased riverine runoff also brings along more nutrients, impacting the eutrophication status of the Baltic Sea (Leppäranta & Myrberg, 2009). The Baltic Sea has been affected by severe eutrophication since the 1960s (Andersen et al., 2016; Cederwall & Elmgren, 1980). Due to a large increase in the production of organic matter and the subsequent increased oxygen demand during decomposition of the produced biomass, the Baltic Sea is also exposed to large‐scale hypoxia (Andersen et al., 2016; Conley et al., 2007). An increase in hypoxia due to rising temperatures and accelerated eutrophication in the Baltic Sea is predicted (Carstensen et al., 2014; Conley et al., 2009; Karlson, Rosenberg, & Bonsdorff, 2002).

Environmental changes, such as warming and decrease in salinity, have been shown to affect zooplankton body size negatively (Daufresne, Lengfellner, & Sommer, 2009; Mäkinen, Vuorinen, & Hänninen, 2017). Elevated temperature can also extend the season by postponing its end (Tachibana, Nomura, & Ishimaru, 2019), shown as cladocerans producing their resting eggs later due to warming (Chen & Folt, 1996). Fish larvae and their recruitment to the population can be especially impacted if the quality of their food community, that is, zooplankton decreases substantially (Arula, Ojaveer, & Klais, 2014; Edwards & Richardson, 2004). Various aspects of zooplankton dynamics have been studied in the Gulf of Riga: the effect of milder winters on small‐sized copepods (Klais, Norros, Lehtinen, Tamminen, & Olli, 2017; Klais, Otto, Teder, Simm, & Ojaveer, 2017), the long‐term abundance dynamics (Ikauniece, 2001), as well as the use of body weight, and body size of zooplankton as biodiversity indicators (Labuce, Dimante‐Deimantovica, Tunens, & Strake, 2020; Simm, Kotta, & Jänes, 2014).

Functional traits, however, have not been widely used to study zooplankton dynamics in the Baltic Sea. (Pecuchet et al., 2019). The aim of this study is to study how the zooplankton community (hypotheses 1–2: community structure; hypotheses 3–4: functional diversity) is associated with measured environmental variables in the Gulf of Riga using a hypothesis‐driven approach. We expect that decreasing salinity, warming and decreasing oxygen will affect zooplankton negatively. We also expect milder winters to reduce functional diversity (see *Hypotheses*). The long‐term dataset is extensive and can readily be used to reveal patterns, such as changes in functional diversity, occurring in brackish areas as a consequence of environmental change.

## 2 MATERIAL AND METHODS

### 2.1 Study area

The Baltic Sea is a shallow (mean depth 54 m), strongly stratified brackish sea with a topography consisting of several subbasins, and is connected to the North Sea only by narrow straits allowing minimal water exchange with the fully marine North Atlantic Ocean (Leppäranta & Myrberg, 2009). It is characterized by substantial riverine input and steep geographical and seasonal gradients in temperature, salinity, and nutrient concentrations. The salinity in the Baltic Sea is in some areas low and ranges from 2 to 25 psu (Leppäranta & Myrberg, 2009).

The Gulf of Riga is shallow semi‐enclosed water basin of the Baltic Sea. The average water depth of the open part is 26 m and maximum depth >60 m (Berzinsh, 1995). Pärnu Bay, which represents coastal area, is shallow subbasin (depth varies from 7.5 to 23 m) located in the northeastern part of the open Gulf of Riga. The Gulf is relatively shallow; therefore, the water temperature is highly dependent on the air temperature. A seasonal thermocline may occur in the open Gulf in the summer, which reduces water mixing and can lead to a temporary depletion in the concentration of oxygen in the deeper layers. However, oxygen concentration rarely decreases below the 5 ml/L, hypoxia may occur only temporarily during summer thermal stratification in the deepest area (Kotta et al., 2008).

The Gulf of Riga is located at a temperate latitude with mean summer air temperatures close to approximately 20°C and mean winter temperatures approximately −5°C. The coastal area of the gulf can be ice‐covered up to 80 days (Kotta et al., 2008), but often winters can be ice‐free too. Winter conditions are tightly coupled to the Northern Atlantic Oscillation (NAO), and years associated with a positive NAO are characterized by stronger winds and a substantial increase in rainfall (Kotta et al., 2004). Moreover, cold winters alter the seasonal freshwater inflow, producing a spring freshet (Hänninen, Vuorinen, & Hjelt, 2000). In the Gulf, river discharges mix with brackish Baltic Sea water, yielding average surface salinity between 5.2 and 6.4 psu, whereas bottom layer salinity is >7 psu. However, during spring, surface salinities <2 psu can be observed in the river plumes. In “typical” years, water is cold and no seasonal thermocline occurs until May and the water column remains fully mixed. When the water temperature rises up to about 17–20°C, a thermocline forms, reaching a depth of 25 m in August and disintegrating in September–October due to intensive wind mixing.

### 2.2 Zooplankton monitoring data

**FIGURE 1:**
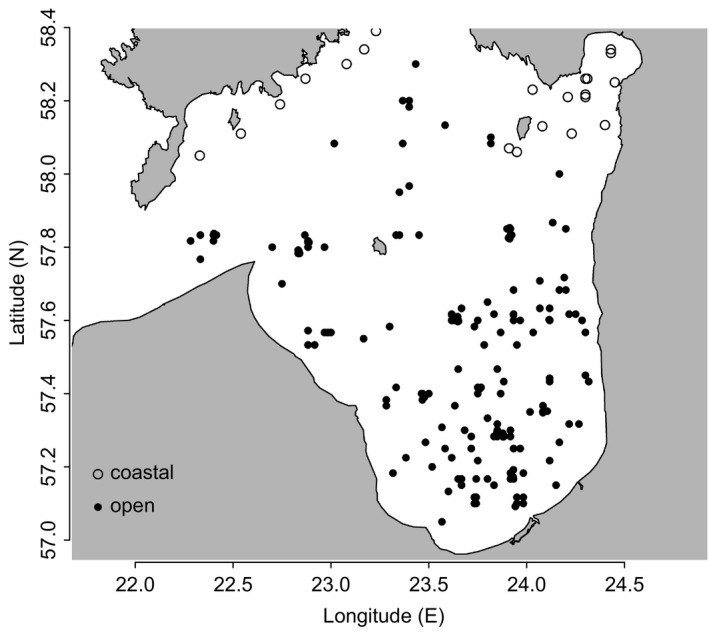
Map of the Gulf of Riga showing the sampling locations; coastal sites are marked with open circles and open sites with black circles

The zooplankton monitoring data from the Gulf of Riga are the most extensive dataset of zooplankton species composition and abundance in the Baltic Sea. Data were collected by the Estonian Marine Institute (UT‐EMI) and Institute of Food Safety, Animal Health and Environment in Latvia (BIOR) since the 1957. The coastal data are collected by UT‐EMI and originate mostly from Pärnu Bay, covering areas between 5 and 15 m deep. Samples were collected with a Juday 90 µm net as a single haul from the bottom (1 m above the sea floor) to surface. Open Gulf data were collected with a Juday 160 net (mesh size 160 µm, mouth diameter ⌀ 36 cm) (UNESCO, 1968) from deeper than 15 m (max depth of open area was 55 m). Sample preparation for counting was performed according to UNESCO (1968). During analysis, zooplankton were identified to species level or to the lowest possible taxonomic level and their number in subsample determined. The number of Copepoda was determined separately for each developmental stage, that is, nauplii NI‐VI, copepodites CI‐V, and adult females and males CVI. The counting procedure in the subsamples was repeated until the number of three dominant species reached 100 individuals. The individuals of large‐sized zooplankton species (*Cercopagis pengoi* and *Limnocalanus macrurus macrurus*) were counted in the whole sample. Finally, the abundance of each species (m^−2^) was calculated from the original abundances (m^−3^) by multiplying this by the sampling depth (Hernroth, 1985). *Cercopagis pengoi* biomass (BIOR data) was calculated using individual weight factor 0.4 (Ojaveer, 1997). Data covered the time period from 1957 to 2012. Coastal sampling was conducted more frequently (in every 2–3 days from 1970 until 1990, and weekly after 1990), and open sea data were collected usually during a sampling campaign once a month (exact week also varied, from beginning to end of month). Major data caps occurred in the coastal data in August before 1970. Since seasonal data for May and August were most consistently sampled both in the coastal and open Gulf areas, the data from these two months were used in the analysis, consisting in total of 2,033 profiles (855 coastal, 1,148 open, 1,048 in May, 985 in August) (Figure 1).

### 2.3 Trends in functional group richness and trait composition

The simplest approach to defining functional diversity is the *functional group richness* (FGR). To calculate the FGR for the samples, species were divided into basic *functional groups*. Functional groups were defined by combining higher level taxonomic classification (mostly Class) with the feeding mode. The prominent groups Cladocera and Copepoda were further classified into “large” (e.g., copepods Pseudocalanus spp., Limnocalanus macrurus *macrurus*, Centropages hamatus, cladocerans Daphnia galeata, Cercopagis pengoi) and “small” (e.g., copepods Eurytemora affinis, Acartia spp., cladocerans Bosmina spp., Evadne nordmanni, Podon polyphemoides) (see also Klais et al., 2016). Values of a relatively simple set of functional traits were assigned to more frequently encountered zooplankton taxa (species or higher) in order to analyze the shifts in functional composition of communities in further detail (Table 1).

**TABLE 1:** Functional traits used in this paper, and organization of trait table

Taxa/Functional traits	Complexity	Filtering	Predation	Min. length (mm)	Max length (mm)
*Eurytemora affinis*	3	1	0	0.35	1.25
Copepoda	3	1	0	0.4	1
*Acartia* sp.	3	1	1	0.29	1.3
*Bosmina (Eubosmina) coregoni*	2	1	0	0.15	0.8
*Synchaeta baltica*	1	1	0	0.19	0.35
*Keratella* sp.	1	1	0	0.13	0.18
*Amphibalanus improvisus*	4	1	0	0.15	0.43
Bivalvia	4	1	0	0.15	0.4
*Podon polyphemoides*	2	0	1	0.2	0.7
*Evadne nordmanni*	2	0	1	0.25	1
*Limnocalanus macrurus macrurus*	3	1	1	0.6	3

### 2.4 Principal coordinate analysis to reveal functional (dis)similarities between taxa

After the assembly of the functional trait table, principal coordinate analysis (PCoA) of the species functional traits was used to construct a “functional trait map” from the 204 taxa with complete set of trait information. Distance matrix for PCoA was calculated with Gower distance that allows different trait types (continuous, binary, categorical) to be combined. Minimum and maximum length was log transformed before calculating the distance. The first two principal coordinates were kept in PCoA to create a 2D representation of the functional differences between species.

### 2.5 Long‐term shifts in the functional composition of communities

After the taxa had obtained coordinates in the PCoA, the functional composition was described by the position of the community in the PCoA plot. The position of the community was calculated as a mean position of the taxa in the sample with respect to A1 and A2, weighted by their relative abundances. Long‐term trends (explanatory variable year) were then visualized and quantified by generalized additive model (GAM) smooth functions (family Gaussian, link identity), using the community coordinates A1 or A2 in May or August as response variables. Models were fitted to A1 and A2 separately, since it is more difficult to visualize and interpret the temporal trends on a 2D surface. To analyze the trends in A1 and A2 independently was also meaningful, because the distribution of trait values along A1 and A2 is straightforward (feeding type varies along A1, complexity and body size vary along A2).

### 2.6 Environmental drivers and zooplankton functional composition

The environmental variables salinity, temperature, and turbidity, as well as proxies of eutrophication (chlorophyll *a*, and dissolved oxygen), were not measured consistently during the monitoring cruises. The model data are therefore considered a realistic and a more accurate (without measurement error) estimate of salinity, oxygen, and temperature. Oxygen, temperature, and salinity data originate from the 3D coupled sea ice–ocean model for Baltic Sea (BSIOM; Lehmann, Hinrichsen, Getzlaff, & Myrberg, 2014), which has a high spatial resolution (2.5 × 2.5 grid cells) and a 3 m depth resolution for the period of 1979–2014. Oxygen concentration was extracted from the model for the bottom layer (the lowermost 3 m layer), because hypoxia, if present, is most severe at the bottom, as a result of oxygen consumption of settled organic material. Salinity and temperature were calculated as average values of the water column, matching the zooplankton observations (latitude, longitude, month). Winter air temperatures were obtained from three weather stations in the northeastern part of the Gulf of Riga (Pärnu, Kihnu and Sõrve) and averaged to daily mean values. Winter harshness was calculated as a sum of the negative daily air temperatures between November and April. To investigate the potential connections between environmental variables and zooplankton community composition, we used a hypothesis‐driven approach.

#### 2.6.1 Hypothesis 1: Decreasing salinity promotes rotifers and cladocerans

We expected the share of rotifers and cladocerans to be negatively affected by salinity (Gutierrez *et al*., 2018). To find support for H1, we looked for expected links between the annual variations in community composition. In particular, we looked in to the average position along the A2‐axis that corresponded to the complexity, or the relative share of rotifers and cladocerans in summer as a function of annual mean salinity in the study area.

#### 2.6.2 Hypothesis 2: Higher summer water temperature promotes smaller organisms

We expected the community to change toward the dominance of smaller organisms with increasing temperature (Daufresne et al., 2009). H2 was tested similarly to H1, using water temperature as the predictor.

#### 2.6.3 Hypothesis 3: Milder winters and higher water temperature in May lead to higher functional diversity (phenological shift)

The hypothesis that milder winters bring higher functional diversity in May was based on two assumptions or observations: (1) Phenological shifts occur with milder winters, and the communities typical for summer will appear earlier, and (2) during the summer, the communities are usually functionally more diverse (Richardson, 2008). To test H3, FGR was analyzed as a function of winter air temperature and water temperature in May.

#### 2.6.4 Hypothesis 4: Lower oxygen concentration in summer will have a negative effect on the functional diversity

Lower oxygen content in summer refers to when dissolved oxygen content decreased and oscillated between 5 and 6 μg/L, and was expected to reduce the functional diversity. H4 was expected to be true in the open gulf; therefore, only open Gulf data were used.

### 2.7 Sensitivity tests

To ascertain that none of the long‐term changes were caused by sampling or by taxonomic artefacts, sensitivity analyses were carried out, in which the extent of the repositioning of communities at the PCoA space was assessed while omitting one taxon at the time from the data. This analysis was done by the same subsets of data as shown in Figure 4. Diagnostics plots were drawn for the most influential taxa (Fig. S1) that were selected after omitting a particular taxon. The A1 or A2 values of the selected samples had a Pearson's correlation with full community A1 or A2 values <0.9). No artefacts (i.e., sudden unexplainable appearance or disappearance of the taxon) were revealed during this test, although several taxa had a strong impact on the long‐term trend. Altogether 11 influential taxa were identified during this test in 19 cases, and in 5 cases, a long‐term trend became notably stronger after excluding a single taxon (e.g., *Synchaeta* sp. and *Acartia* sp. in open sea area in May along PCoA axis 1 (Fig. S1c), or *Bosmina* (*Eubosmina*) *coregoni*. in open sea in August, along PCoA axis 1, Fig. S1g). All analyses were performed using the R software (version 3.4.0) and RStudio (version 1.1.442; R Core Team, 2020). Generalized additive models were fitted with the package mgcv (version 1.8‐20, Wood, 2017), PCoA was done with the package ade4 (1.7‐6) (Dray & Dufour, 2007), and Gower distance between species from functional traits was calculated with package FD (1.0‐12) (Laliberté & Legendre, 2010).

## 3 RESULTS

### 3.1 Taxonomic composition of the Gulf of Riga zooplankton

**FIGURE 2:**
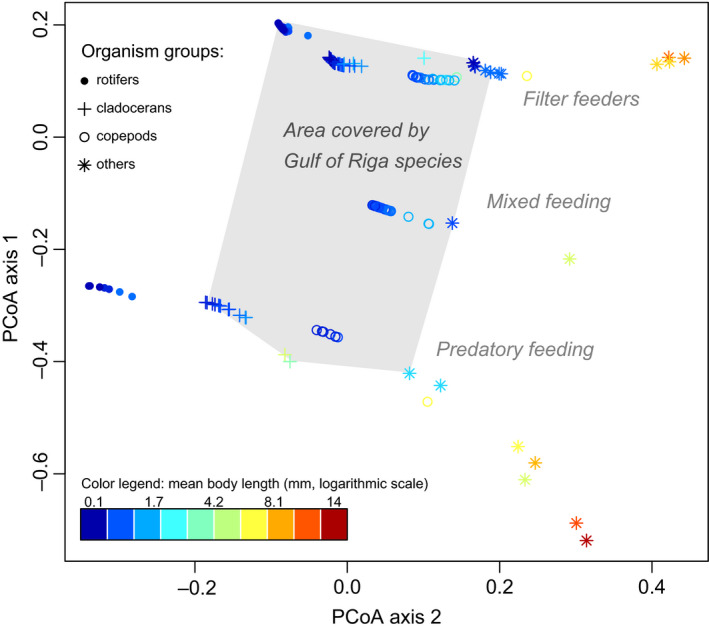
Principal coordinate analysis (PCoA) of the functional trait values of Baltic Sea zooplankton. Colored points correspond to individual taxa (species or higher, *n* = 204). The effect of different traits on the PCoA results is visualized by symbols and color:Color describes the mean body length (middle point between the minimum and maximum body size during life cycle); symbols indicate the large grouping of organism (four categories: rotifers, cladocerans, copepods, and others). Light gray polygon is a convex hull defined by the subset of taxa that were found in the Gulf of Riga. Along the PCoA axis 1, the species were separated by the feeding type: Values above the 0 belong to the filter feeding organisms, mixed feeding type is found between the 0 and −0.2, and all points below −0.2 are the predators

Altogether 38 taxa occurred in the data, of which 22 were present in >5% of the profiles. In coastal areas (970 profiles), the top taxa (based on frequency of occurrence in profiles) are Copepoda (mostly nauplial stages), *Acartia* spp., *Eurytemora affinis*, *Keratella quadrata*, *Synchaeta baltica*, *Bosmina (Eubosmina) coregoni*, Bivalvia, *Synchaeta monopus,* and *Amphibalanus improvisus* (Table 1). In the open Gulf (1,148 profiles), the respective list of taxa is *Acartia* sp., *E. affinis*, *Synchaeta* sp., *Limnocalanus macrurus macrurus*, *B. coregoni, Keratella* sp., *Evadne nordmanni*, *Pleopis polyphemoides*, *Cyclops* sp., and Bivalvia. Copepods that feed by filtering or have a mixed feeding type and filtering rotifers were the three functional groups present in nearly all samples (Figure 2).

### 3.2 Long‐term trends in FGR

**FIGURE 3:**
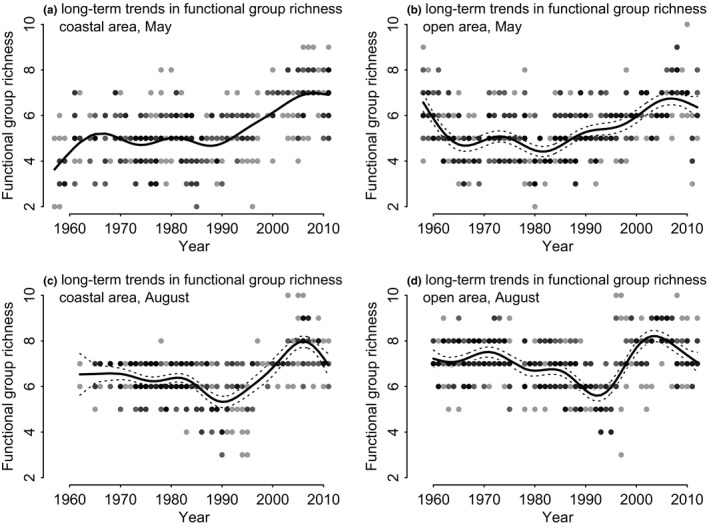
Long‐term trends in FGR during 1957–2012 in the coastal areas (a, c), and in the open Gulf (b, d). Each dot represents the number of functional groups per sample, and the lines are respective smoothed curves against year (i.e., average)

During the investigated time period, the FGR varied between the season and area (Figure 3). In the 1960s, the spring time (May) FGR exhibited opposite patterns in the coastal and open sea areas. In the coastal area, FGR was initially lower (ca. 4) and increased during the decade, whereas in the open sea the FGR initially decreased from ca. 7 to 5. After this time period, FGR followed similar patterns. In the coastal area, a steep increase in FGR has occurred since the 1990s, and in the open sea area, FGR has increased gradually since the 1980s.

In August, FGR showed similar patterns in both coastal and open sea areas. Initially, late summer FGR decreased until the beginning of 1990s and was at its lowest in 1990, whereafter it increased in both areas until the early 2000. Since the peak in the mid‐2000, a decrease has been recorded. In both areas, FGR was always higher in late summer than in spring.

### 3.3 Long‐term shifts in trait composition

**FIGURE 4:**
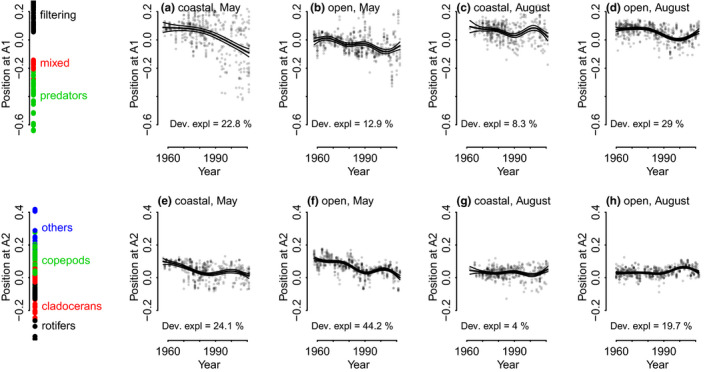
Long‐term shifts in the position of communities with respect to functional trait map first (A1, upper row) and second coordinate (A2, lower row), in the coastal and open Gulf in May and August between 1957 and 2012

The significance of long‐term variability was judged from the deviance explained by the GAM curves. Only the coastal community in August did not change over time, when judging from the GAM fit on A1 and A2 as a function of year (Figure 4c,g). In all other cases (open Gulf in August, and both open and coastal areas in May), pronounced long‐term variability occurred both in A1 (mostly reflecting the feeding type) and in A2 (mostly reflecting the complexity).

The most obvious trend in functional composition was the shift toward negative values of A1 indicating a shift from filtering organisms to mixed feeders and predators in the open sea late summer communities 1980‒2000 (Figure 4d; August). This trend, however, reversed in early 2000 simultaneously with a shift toward more complex organisms (until 2000). Also, in both areas in May (Figure 4a,b), the long‐term trend was toward increasing share of mixed or predatory feeding type.

Significant long‐term variability was also evident in A2 values. In the spring communities (May), the prominent long‐term shift in complexity was toward higher share of simpler organisms (Figure 4e,f). In August, the functional composition of both coastal and open area communities remained almost unchanged.

### 3.4 Long‐term trends in the main organism groups

**FIGURE 5:**
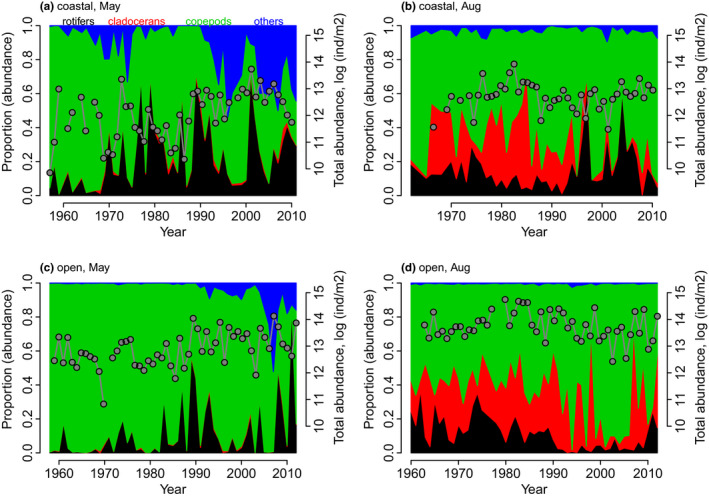
Annual mean length‐adjusted abundance proportions of the main organism groups (rotifers = black, cladocerans = red, copepods = green, others = blue) and total length‐adjusted abundance of the zooplankton community (gray points)

We compared the mean proportions of each groups’ abundance (rotifers, cladocerans, copepods, others) to the total abundance of organisms in order to detect patterns in the long‐term dynamics of the main groups. In the coastal area in May (Figure 5a), the proportion of rotifers increased from 1960s onwards from ca. 40% to ca. 70% of the whole community abundance; however, the proportion varied strongly over time. In the 1960s, copepods were dominating the community. Since then a large decrease in the abundance of copepods has been recorded in connection to an increase of rotifers and, more recently, an increase in the abundance of, for example, bivalves (“others”). Later in the summer (August) in the coastal area, the most notable change was the decrease in the proportion of cladocerans in the mid‐1980s to very low abundances in the mid‐1990s and beyond (Figure 5b). After the mid‐1990s, rotifers accounted for >50% of the organisms recorded. In the open sea area in May (Figure 5c), the community was mostly dominated by copepods until the 1980s, whereafter the proportion of rotifers increased and dominance relations started to shift from copepods to rotifers. Toward the end of the investigated time period, from ca. 2005, the proportion of copepods decreased by ca. 50%, related to the increased proportion of “others.” In August in the open sea area (Figure 5d), the large decrease of rotifers from 1990s to 2010 gave rise to increasing dominance of cladocerans and copepods.

### 3.5 Trends in relevant environmental drivers

**FIGURE 6:**
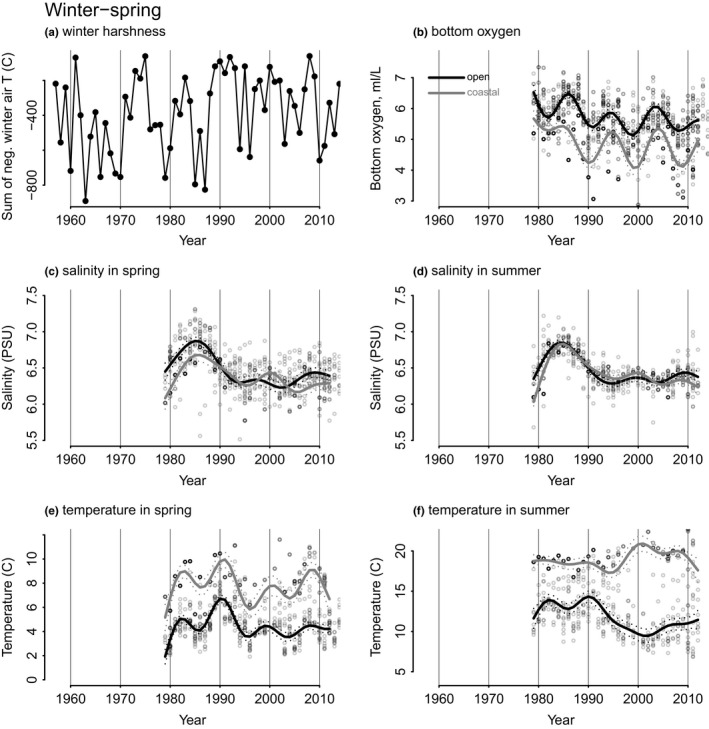
Long‐term trends in the environment: (a) winter air temperature (sum of negative temperatures); (b) bottom oxygen concentration in late summer/August; (c,d) salinity in May and August; and (e,f) temperature in May and August. In figures (b–f), the open sea area is denoted with black line, and the coastal area with gray line. The data are derived from model data

Winters were generally harsh, but more variable before the 1990s. The winters 1990–1995 were relatively mild, and since then gradual cooling has occurred (Figure 6a). The winter air temperatures largely follow the Baltic Sea index. Oxygen concentration was variable, yet slightly declining during the entire period 1978–2012, and no hypoxic or anoxic levels were estimated (Figure 6b). Salinity peaked in the mid‐1980s both in spring and summer, and in open and coastal areas, whereafter it declined until the mid‐1990s and has fluctuated slightly since (Figure 6c,d). In the spring, surface temperature fluctuated more until mid‐1990s in both open and coastal sea areas, whereafter it was slightly increasing in the coastal area (Figure 6e).

### 3.6 Functional composition and environmental factors

#### 3.6.1 Hypothesis 1: Salinity

**FIGURE 7:**
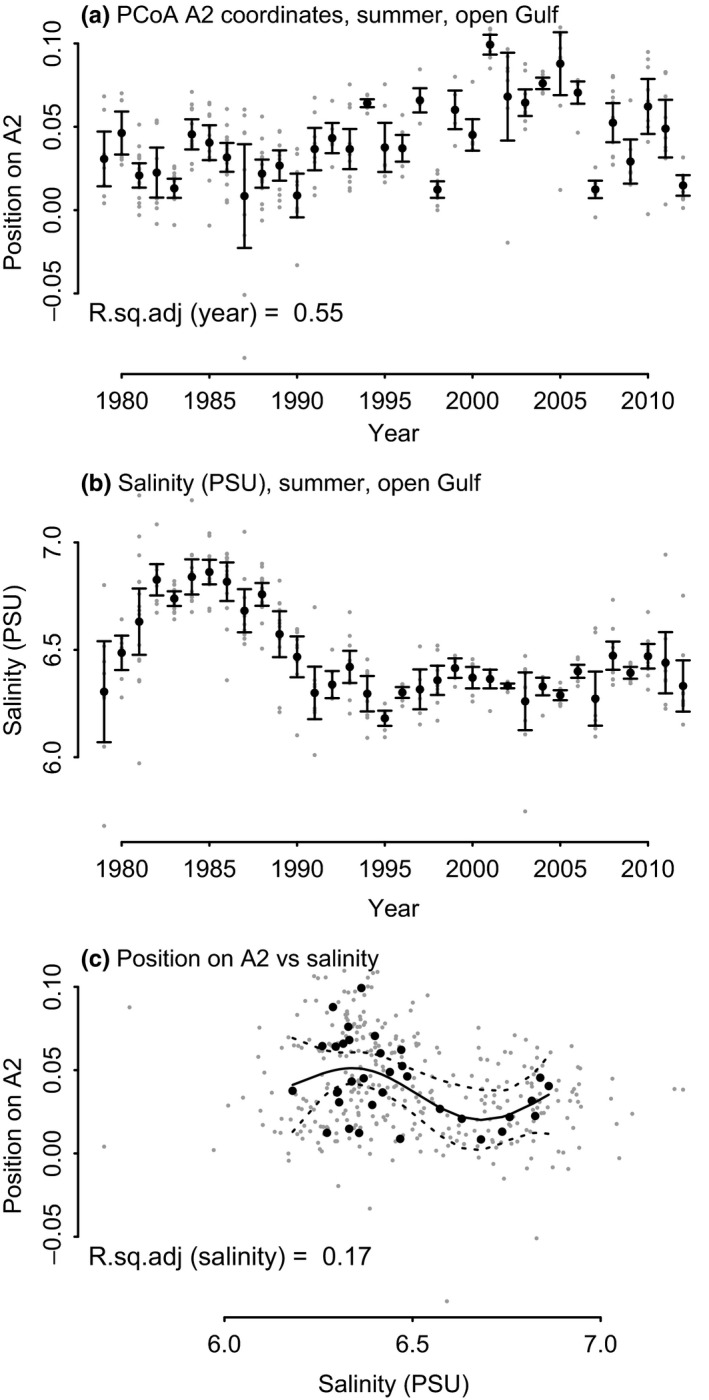
The community composition as the position on the PCoA axis A2 (a) and its relation to salinity (b and c). In (a) and (b), gray dots are individual samples and the black dots are annual means, and the bars indicate 2*standard error of the mean. In (c), gray dots are individual samples, black dots are annual means, the solid line the GAM fitted curve, and the dashed lines represent 95% confidence interval of the fit

**FIGURE 8:**
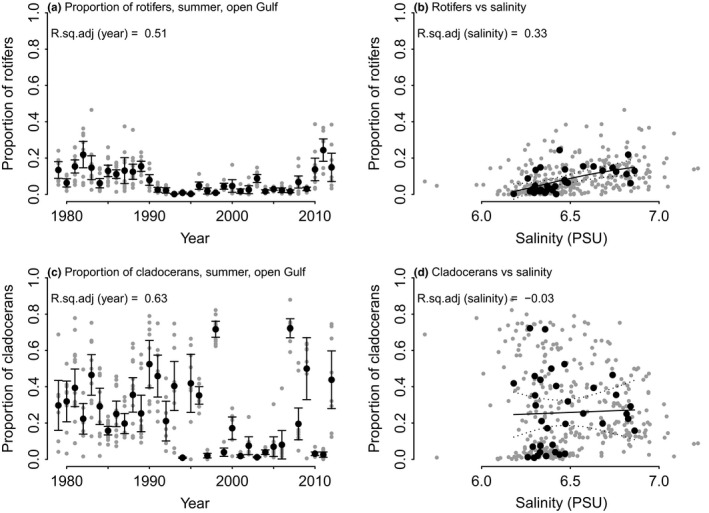
The effect of salinity on the main groups of interest: rotifera (a and b) and cladocera (c and d). In (a) and (c), gray dots are individual samples, black dots are annual means, and the bars indicate 2*standard error of the mean. In (b) and (d), gray dots are individual samples, black dots are annual means, the solid line the GAM fitted curve, and the dashed lines represent 95% confidence interval of the fit

The share of cladocerans and rotifers was expected to be negatively affected by salinity. This hypothesis was, however, not supported. A negative link was found between salinity and A2 axis of PCoA, implying the dominance of simpler organisms as salinity increased (Figure 7c). When looking at the proportions of individual groups, we found the proportion of rotifers to be positively correlated with salinity (Figure 8b). Proportion of cladocerans was highly variable, but there was no link to salinity (Figure 8d).

#### 3.6.2 Hypothesis 2: Temperature

**FIGURE 9:**
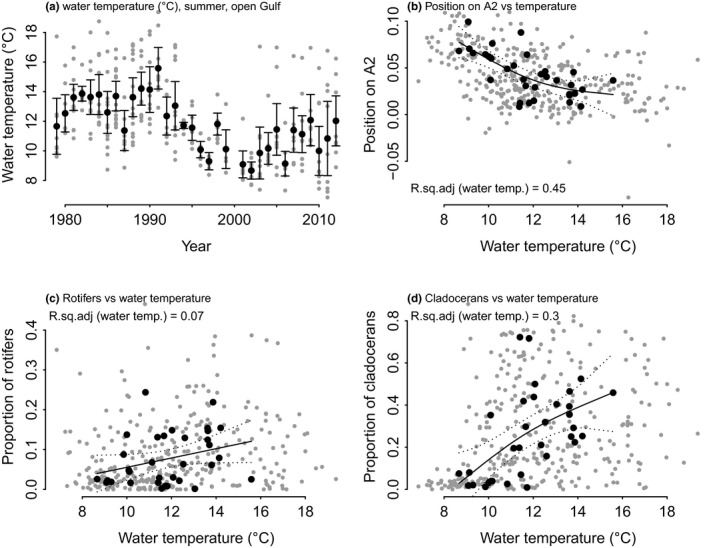
The effect of water temperature (a) on the community composition, and the (b) position on the PCoA plot axis A2, and the proportion of rotifers (c) and cladocerans (d) vs. water temperature during 1979–2012. In figures (a) and (c), gray dots are individual samples, black dots are annual means, and the bars indicate 2*standard error of the mean. In (b) and (d), gray dots are individual samples, black dots are annual means, the solid line the GAM fitted curve, and the dashed lines represent 95% confidence interval of the fit

The community was expected to change toward dominance of smaller organisms with increasing temperature. This hypothesis was supported by the shifts in the community. The negative link between temperature and community position along A2 axis of PCoA implied on average lower complexity of organisms with warming (Figure 9b). Looking only at groups, the relative proportion of cladocerans was particularly responsive to mean temperature (Figure 9d).

#### 3.6.3 Hypothesis 3: Winter harshness

**FIGURE 10:**
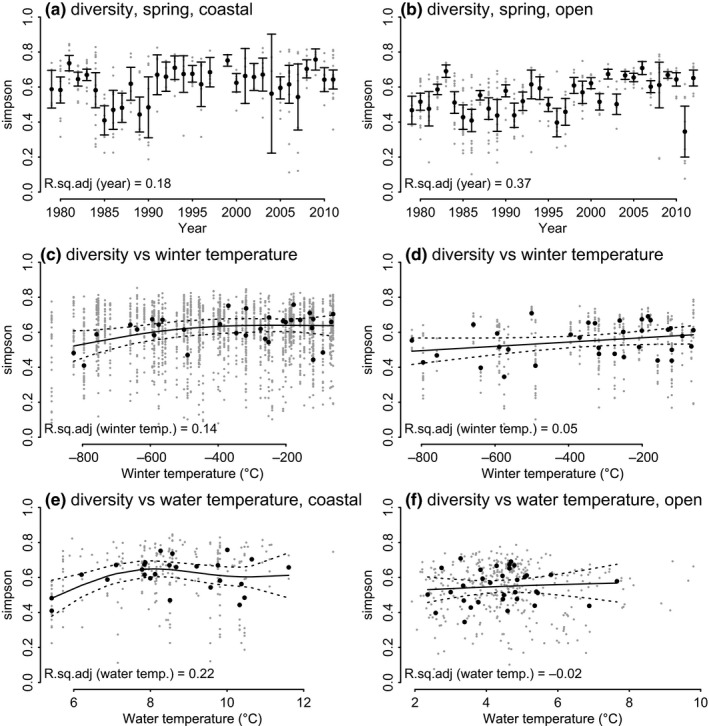
Functional diversity (fgH) in the spring (May) in the coastal (a) and open (b) sea areas, and its relation to winter air temperature in coastal and open sea areas (c and d, respectively) and water temperature in the spring (e and f) during 1979–2012

Milder winters were expected to contribute to higher functional diversity (H) in May. The data provided only modest support to this hypothesis; as in the open sea area, the higher functional diversity was observed with warmer winters (Figure 10d). In coastal sea, the link with winter air temperature was unimodal; highest during “average” winters (Figure 10c). Correlation between the functional diversity and water temperature was not consistent with our expectation: negative in coastal area, unimodal in open Gulf (Figure 10e,f).

#### 3.6.4 Hypothesis 4: Oxygen

**FIGURE 11:**
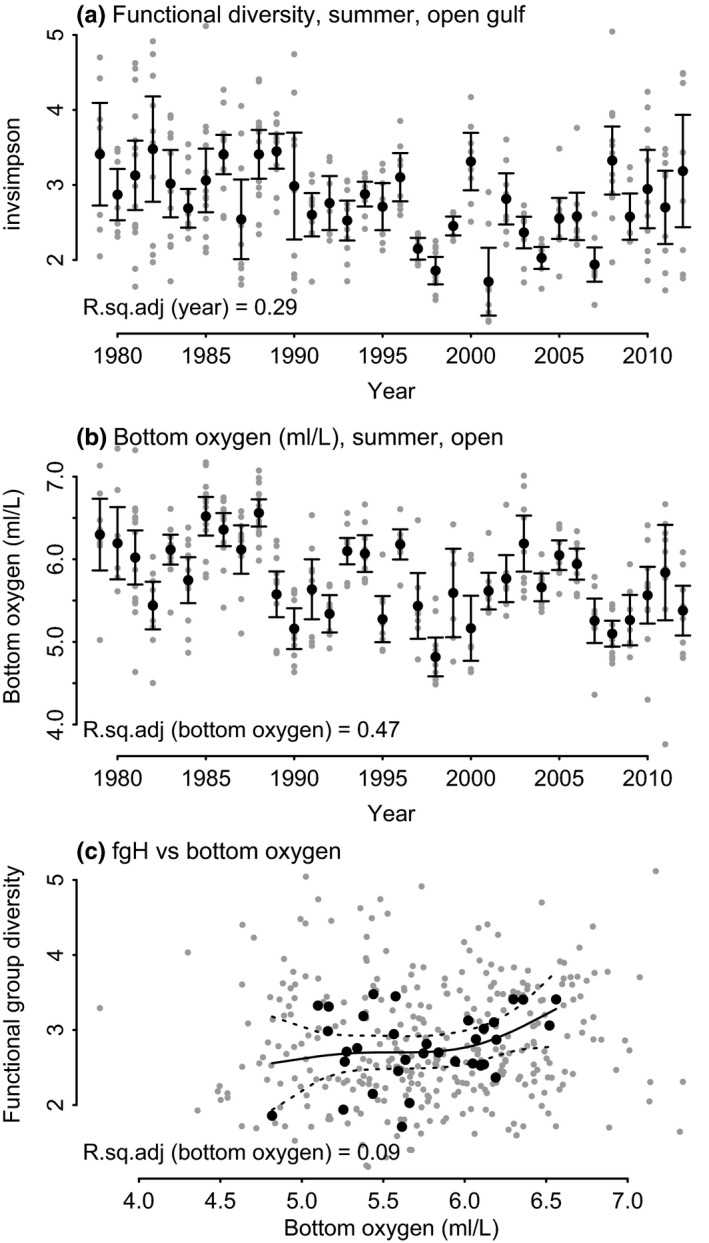
Functional diversity (fgH) in August in the open sea area (a), bottom oxygen (b), and functional diversity relation to bottom oxygen concentration (c)

Lower oxygen content (4–5 μg/L, see Figure 6b) in late summer was expected to reduce the functional diversity (H); however, only a weak link between H and oxygen concentration near the sea floor was found (Figure 11c).

## 4 DISCUSSION

In this paper, we investigate changes using functional traits and groups that have taken place in the Gulf of Riga zooplankton communities during the past five decades in relation to environmental variables. One of the most significant changes was the considerable increase of the zooplankton functional groups (FGR) in coastal springtime communities, and dominance shifts in functional trait composition.

### 4.1 FGR and functional trait composition

In the spring, the FGR of zooplankton increased overall during the investigated time period 1960–2010; in the coastal area, the increase was nearly twofold. Several environmental factors caused the increase of the FGR in the springtime community. We found that the changes in salinity and temperature mainly impacted the community by increasing the abundance of simpler smaller organisms. The higher nutrient load in the system in early spring increased the amount of functional groups. In the late summer, the FGR gradually decreased from the 1970s and 1980s both in the open sea area and coastal area, until the 1990s where the richness was at its lowest. This most likely reflects the system response to eutrophication that has been extensive in the Baltic Sea since the 1960s (Andersen et al., 2016; Cederwall & Elmgren, 1980).

Regarding functional trait composition, the main long‐term shift that occurred during 1960–2010 was the shift from a community dominated by filterers in the 1960s toward a mixed‐ and predator‐dominated community in the early 2000. Also, the complexity of the organisms changed from more complex groups (Copepoda) to a community dominated by cladocerans and rotifers. Rotifers are known to increase in murky lakes (Leech, Pollard, Labou, & Hampton, 2018); they also respond to warming (Daufresne et al., 2009) and to eutrophication (Vehmaa, Katajisto, & Candolin, 2018). The Gulf of Riga is one of the most eutrophicated basins in the Baltic Sea (HELCOM, 2018), which is a likely reason for the strong increase in rotifers. Vehmaa et al. (2018) showed that small‐bodied cladoceran such as *Bosmina* (*Eubosmina*) *coregoni* also respond to eutrophication by increasing their abundance. Even though we could not include phytoplankton data in the current paper, numerous studies have shown how microalgal communities have changed during the past decades in the Baltic Sea. Cryptophytes have decreased, whereas cyanobacteria, chrysophytes and chlorophytes have increased (Kuosa et al., 2017; Suikkanen, Laamanen, & Huttunen, 2007), which most likely has a strong effect on the grazer communities by causing changes in food quality.

### 4.2 Zooplankton community structure

The large decrease in the proportion of copepods in both coastal and open sea springtime communities was among the most notable changes that took place in the zooplankton community structure. Supporting evidence has been found in the Gulf of Finland (Suikkanen et al., 2013) and the Gulf of Bothnia (Kuosa et al., 2017). The decrease was most likely occurring due to the decrease in the dominant taxa *Acartia* spp. and *E. affinis* and their nauplii*. E. affinis* is considered a euryhaline species, with a wide salinity tolerance, whereas *Acartia* has a marine origin (Dutz & Christensen, 2018). Casini et al. (2012) reported that herring abundances were inversely correlated with copepod abundances in the Gulf of Riga, suggesting top‐down control may be important in the area (cf. Pecuchet et al., 2019).

The reason to the findings has been suggested to be rapid warming that is affecting animal size negatively (Daufresne et al., 2009). Overfishing could be another factor indirectly affecting copepod populations, by causing diminishing of cod populations, and thereby enabling planktivorous fish populations such as Baltic herring *Clupea harengus harengus* and sprat *Sprattus sprattus* to flourish (Casini et al., 2008; Ljunggren et al., 2010). Eutrophication can also affect copepod populations negatively via decreased food quality caused by harmful algae blooms, which have become more frequent in the Baltic Sea and globally (O'Neil, Davis, Burford, & Gobler, 2012). The decrease of copepods occurred simultaneously with the increasing proportion of rotifers and more complex organism groups, named “others.” Especially in springtime in the coastal area, the peak of “others” since ca. 1995 was likely mostly attributed to the appearance and rapid increase of *Marenzelleria* spp. larvae that are abundant in the water column from early spring (Kauppi, Norkko, & Norkko, 2018). *Marenzelleria* first appeared in the southern Baltic Sea in 1985 (Bick & Burckhardt, 1989) and has since then become a dominant component of the benthic ecosystem (Norkko et al., 2015).

### 4.3 Functional composition and environmental factors

We expected the abundance of small‐bodied organisms, that is, rotifers and cladocerans, to increase with decreasing salinity. Interestingly, the abundance of rotifers showed a slight increase in the late summer (August) when salinity increased. The salinity change during the investigated time period was <1 unit, and the natural salinity variation that the organisms experience often exceeds this both seasonally, for example, in spring during the emergence from sediment, and spatially, for example, during diel vertical migration. Most Baltic organisms are euryhaline, and ecosystems, such as the Baltic Sea, where the communities are already exposed to large variability regarding a range of environmental parameters, are often expected to be less impacted by future changes as the organisms are adapting to live in such variable conditions. Remane (1934) showed that a salinity of over ~6 psu is an important boundary for animals adapted to brackish water, and some of the copepods live on the edge of their salinity tolerance in the Baltic Sea (Pecuchet et al., 2019), such as *Acartia* spp. (Dutz & Christensen, 2018) and *Eurytemora affinis* (Kuismanen, Forsblom, Engström‐Öst, Båmstedt, & Glippa, 2020).

Temperature can act directly or indirectly on zooplankton community structure; directly, because of the impact it has on the metabolism and reproduction of the organisms, and indirectly via the competition mechanisms when different zooplankton functional groups compete for prey. Increasing temperature brings along a larger share of smaller sized and lower complexity organisms, as is shown, for example, by Vuorinen, Hänninen, Viitasalo, Helminen, and Kuosa (1998), Suikkanen et al. (2013), and Mäkinen et al. (2017). Also, in the Gulf of Riga, the share of lower complexity organisms—most often cladocerans and rotifers—increased simultaneously with temperature rise. The increasing share of low complexity organisms which leads to its dominance instead of larger organisms (copepods) has the potential to decrease the energy availability of higher trophic level organisms, thus causing changes in the food supply chain (Leech et al., 2018). Also, the carbon transport from the sea surface to the bottom will likely be decreased, as copepods are responsible for a large part of the global carbon cycle (Jónasdóttir, Visser, Richardson, & Heath, 2015).

The influence of indirect factors such as changes in food quality, quantity, and predation pressure can have large impacts on the community via changes in environmental parameters. Vehmaa et al. (2018) demonstrated that food quantity (given as chlorophyll *a* and total organic carbon) increased following eutrophication, using sediment core analysis. However, food quality is also altered by eutrophication (O'Neil et al., 2012), as harmful algae blooms are known to increase in frequency, magnitude, and duration (Huisman et al., 2018), also in the Gulf of Riga as shown by Jurgensone, Carstensen, Ikauniece, and Kalveka (2011). For example, both phytoplankton and also microzooplankton biomass and community structure can significantly affect the structure of zooplankton community. Klais, Norros, et al. (2017) and Klais, Otto, et al. (2017) show that especially nitrogen‐fixers and mixotrophs have increased in the current study area, the Gulf of Riga, between spring and late summer.

Based on the model estimations, we did not detect hypoxic conditions during the investigated time period. Overall, hypoxia is a large problem in the Baltic Sea, and in the future, hypoxic conditions are predicted to be even more common both in frequency and in duration following rising temperatures and accelerated eutrophication (Diaz & Rosenberg, 1995; Kabel et al., 2012). The declining oxygen concentration near the seafloor has the potential to reduce the functional diversity (Vaquer‐Sunyer & Duarte, 2008). Via the benthic life stage that many zooplanktonic organisms have, the zooplankton is strongly affected by the conditions near the sea floor. Where oxygen stress occurs, these hypoxic conditions near the seafloor have the potential to disturb the eggs and resting stages of organisms (Lutz, Marcus, & Chanton, 1992). Most species’ resting stages and eggs are tolerant to low oxygen conditions; yet, with oxygen conditions declining further, the share of tolerant species is slowly reduced. For example, the nauplii of copepods can hatch at very low oxygen concentrations (0.3 ml/L), but under these near‐anoxic conditions their development eventually ceases (Katajisto, 2004). Also, reduced diel vertical migration due to low oxygen levels can change the diversity along the oxygen gradient. The oxygen deficiency stress near the seafloor has the potential to weaken a niche by decreasing the share of species that inhabit the deeper layers and thus increasing the share of species that stay on or closer to the surface.

## 5 CONCLUSIONS

The purpose of using functional traits is to characterize organisms by properties that capture essential aspects of diversity, rather than to analyze individual species. In the current paper, we have used traits to determine how the zooplankton community is associated with environmental gradients in a highly eutrophicated gulf. The benefit of looking into traits has revealed systematic changes in the current community—the long‐term decrease of copepods, increase of rotifers, and dominance shift. On the other hand, the proportion of simpler organisms have increased in spring, whereas the proportion of more complex organisms have increased in late summer. Salinity did not have a clear effect on functional traits, which could be due to the fairly small fluctuations in the Baltic Sea salinity. Neither oxygen concentrations affected traits significantly, which may depend on the fact that oxygen was fairly stable in the used time series; planktonic organisms are also very much able to move away from hypoxic areas. Our data also suggest that organisms of low complexity will benefit from the climate‐induced warming of the environment, whereas salinity and oxygen levels seem to have minor effect on the planktonic community in the Baltic Sea, here exemplified by the Gulf of Riga basin.

## CONFLICT OF INTEREST

The authors declare no competing interests.

## AUTHOR CONTRIBUTION

**Anna Jansson:** Writing‐original draft‐Lead, Writing‐review & editing‐Lead; **Riina Klais‐Peets:** Data curation‐Lead, Formal analysis‐Lead, Methodology‐Lead, Visualization‐Lead, Writing‐original draft‐Supporting, Writing‐review & editing‐Supporting; **Evelina Griniene:** Methodology‐Supporting, Writing‐original draft‐Supporting, Writing‐review & editing‐Supporting; **Gunta Rubene:** Methodology‐Supporting, Writing‐original draft‐Supporting, Writing‐review & editing‐Supporting; **Anna Semenova:** Writing‐original draft‐Supporting, Writing‐review & editing‐Supporting; **Aleksandra Lewandowska:** Writing‐original draft‐Supporting, Writing‐review & editing‐Supporting; **Jonna Engström‐Öst:** Conceptualization‐Lead, Funding acquisition‐Lead, Supervision‐Lead, Writing‐original draft‐Supporting, Writing‐review & editing‐Supporting.

## Supporting information

Supplementary Material

## Data Availability

Data used in this study are deposited in the Dryad Digital Repository https://doi.org/10.5061/dryad.jsxksn06t (Jansson et al., 2020).
