# Supplementary material for: Functional shifts in estuarine zooplankton in response to climate variability
Source: Ecol Evol. 2020 Sep 29;10(20):11591–606. doi: 10.1002/ece3.6793 (PMC7593182; doi:10.1002/ece3.6793)
Supplement: Supplementary file 1 — Supplementary Material [file ECE3-10-11591-s001.docx]

**Supplementary material: results of the sensitivity tests**

*Description of the tests*

Sensitivity tests were conducted to evaluate the robustness of the results shown in Figure 3 of the main text: the long-term trends in the community weighted mean position along the PCoA axis 1 and 2.

Sensitivity tests were done in eight subsets (2 areas, 2 seasons, two explanatory variables). Each test consists of a 38-step loop, where at each step 1 species is omitted from data, new position of the community along A1 and A2 axis is calculated, and compared to the original (full community) values by means of Pearson’s correlation.

For every taxon that led to the Pearson correlation <0.9 between the community positions when taxon was excluded, and the full community position, a three- part diagnostics plot was drawn, including: a) the full-community long-term trend (a corresponding panel of Figure 3); b) new long-term trend after the taxon was excluded; and c) long-term abundance trend of the taxon.

Figures will follow in next pages, summary table below lists the taxa that were found influential to the long-term trends observed in Figure 3.

**Table S1.** The taxa that affected long-term trends in community position on functional trait map.

|  | Along the PCoA axis 1 (feeding type) | Along PCoA axis 2 (organism group/size) |
| --- | --- | --- |
| Coastal, May | Polychaeta sp. (Fig. S1a) | *Synchaeta baltica*, *Synchaeta* sp., Copepoda (Fig. S1b) |
| Open, May | *Synchaeta* sp*., Acartia* sp*.,* Polychaeta sp. (Fig. S1c) | *Limnocalanus macrurus, Synchaeta* sp*.* (Fig. S1d) |
| Coastal, August | *Acartia bifilosa, Bosmina* sp., *Keratella cochlearis* (Fig. S1e) | Copepoda spp. (Fig. S1f) |
| Open, August | *Acartia* sp., *Bosmina* sp., *Keratella* sp. (Fig. S1g) | *Eurytemora affinis, Keratella* sp., *Bosmina* sp*.* (Fig. S1h) |


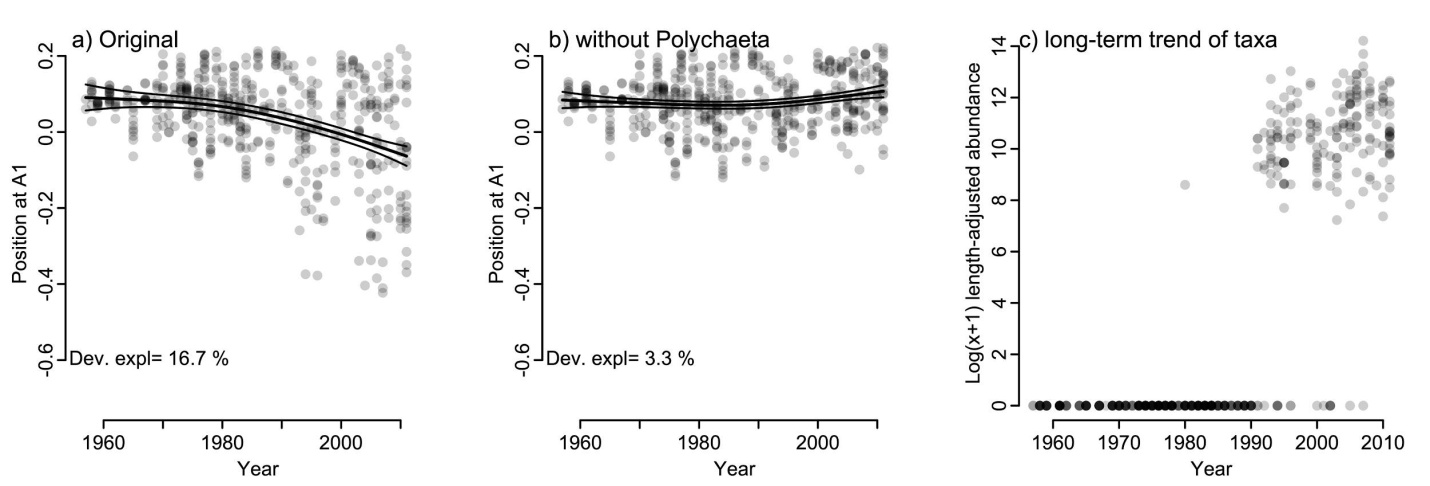


Figure S1a. Coastal, May; position along PCoA axis 1.


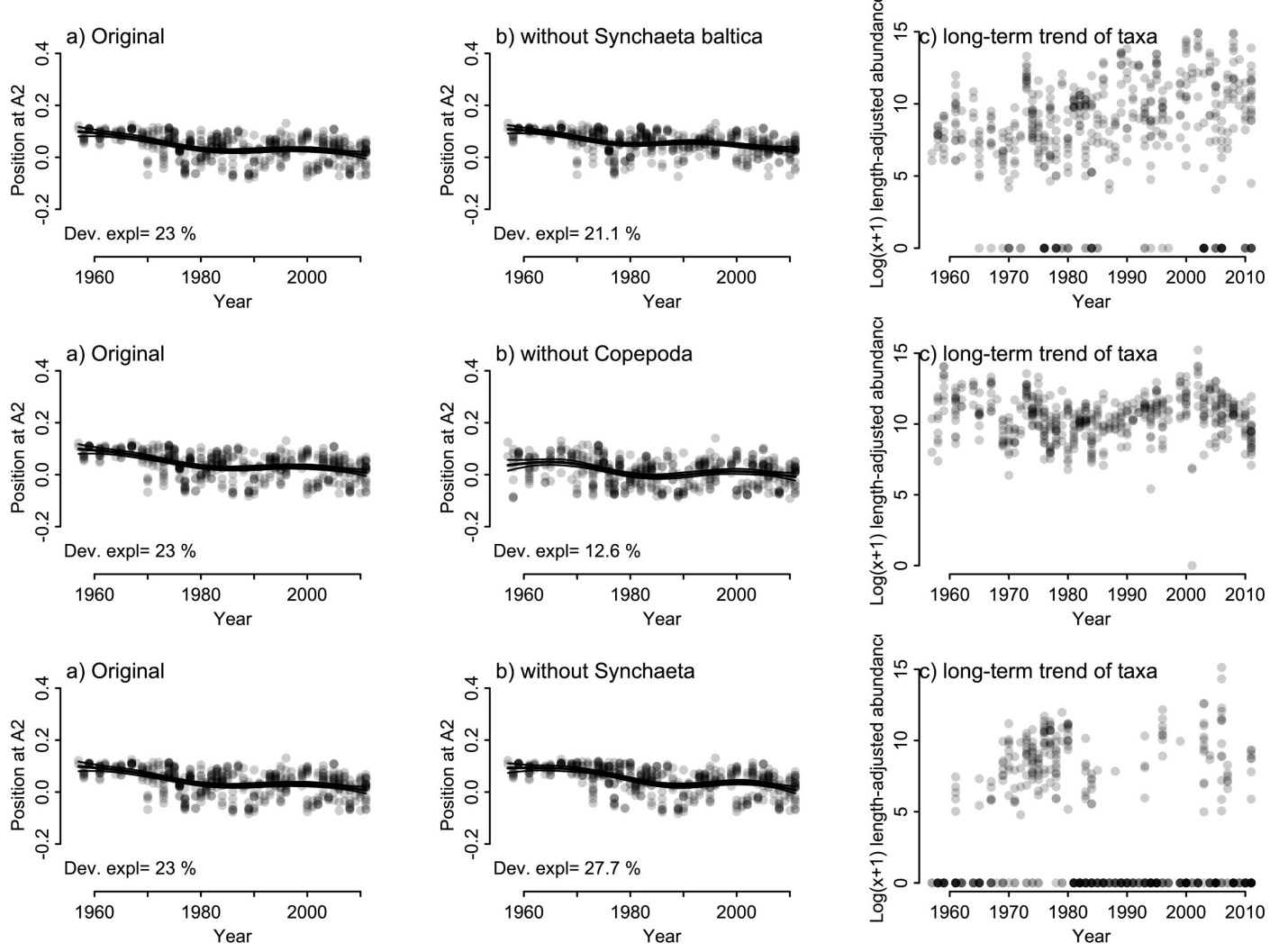


Figure S1b. Coastal, May; position along PCoA axis 2.


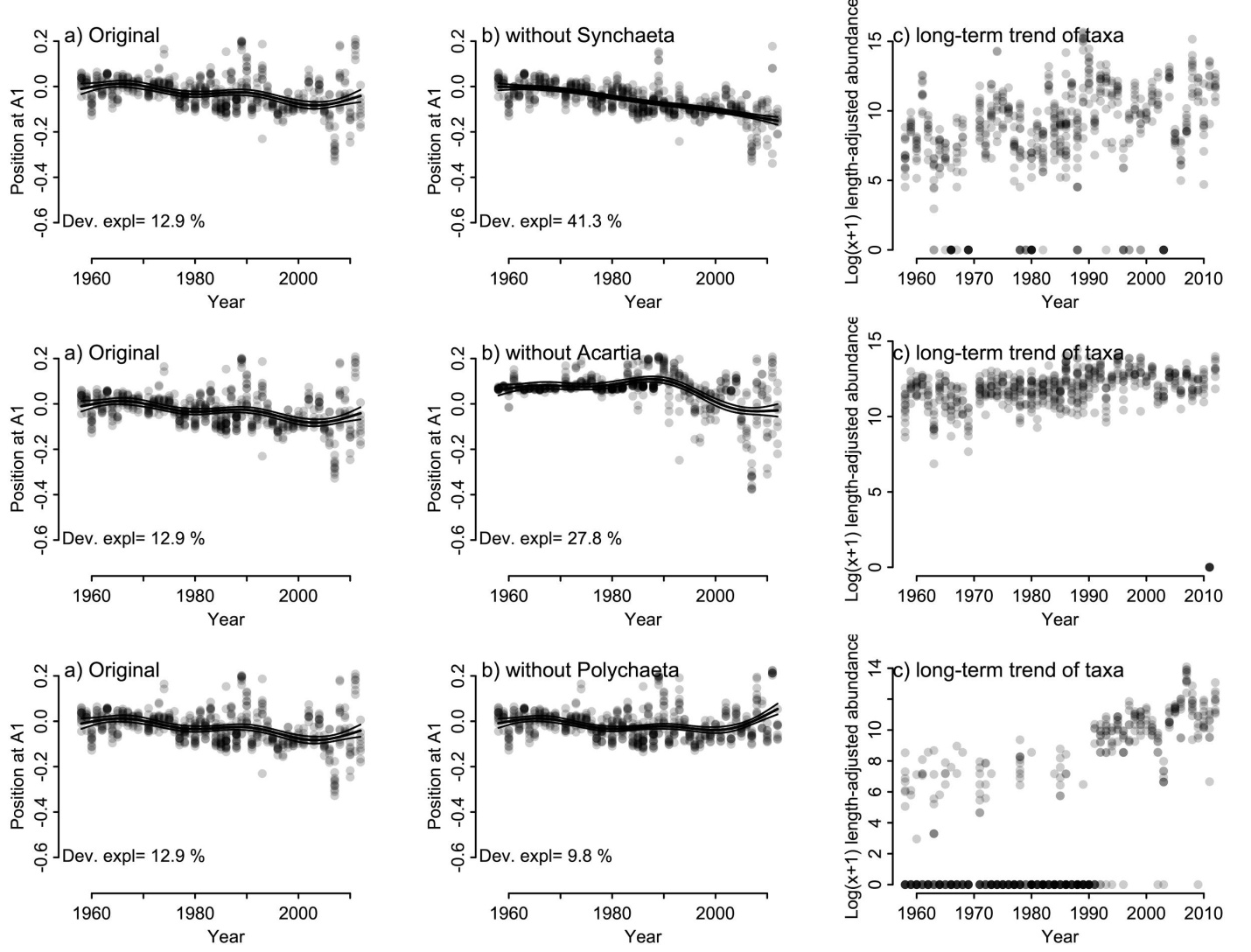


Figure S1c. Open, May; position along PCoA axis 1.


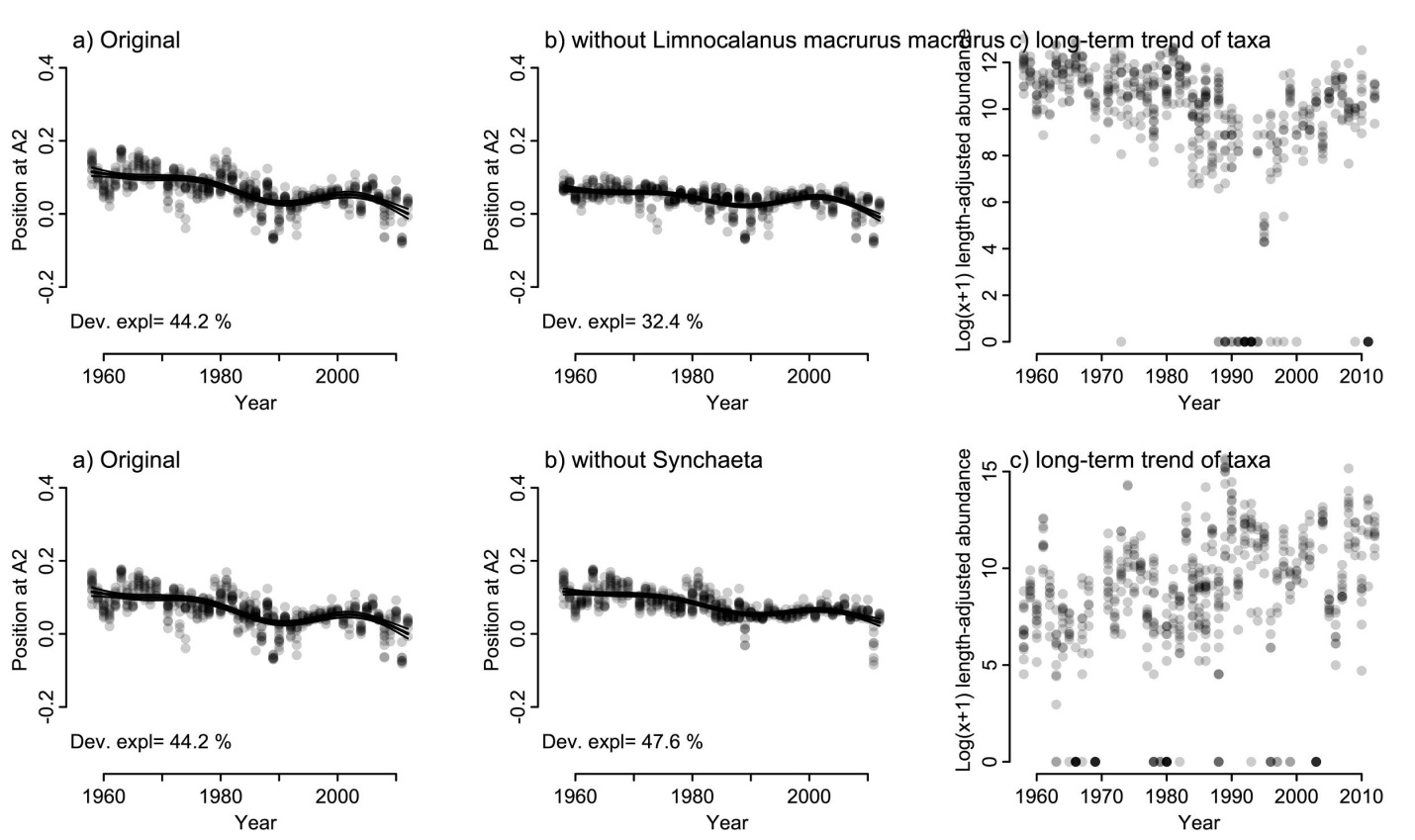


Figure S1d. Open, May; position along PCoA axis 2.


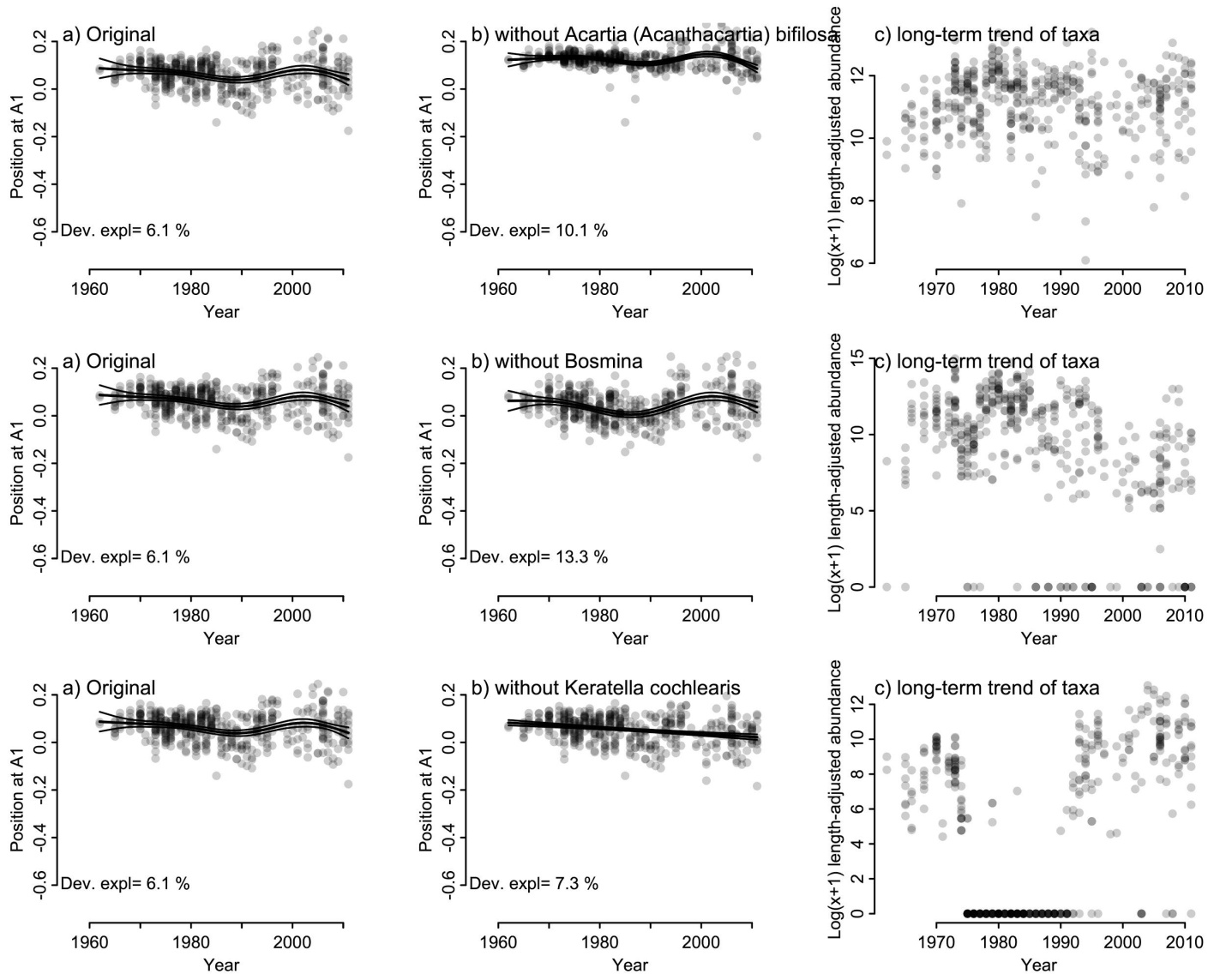


Figure S1e. Coastal, August; position along PCoA axis 1.


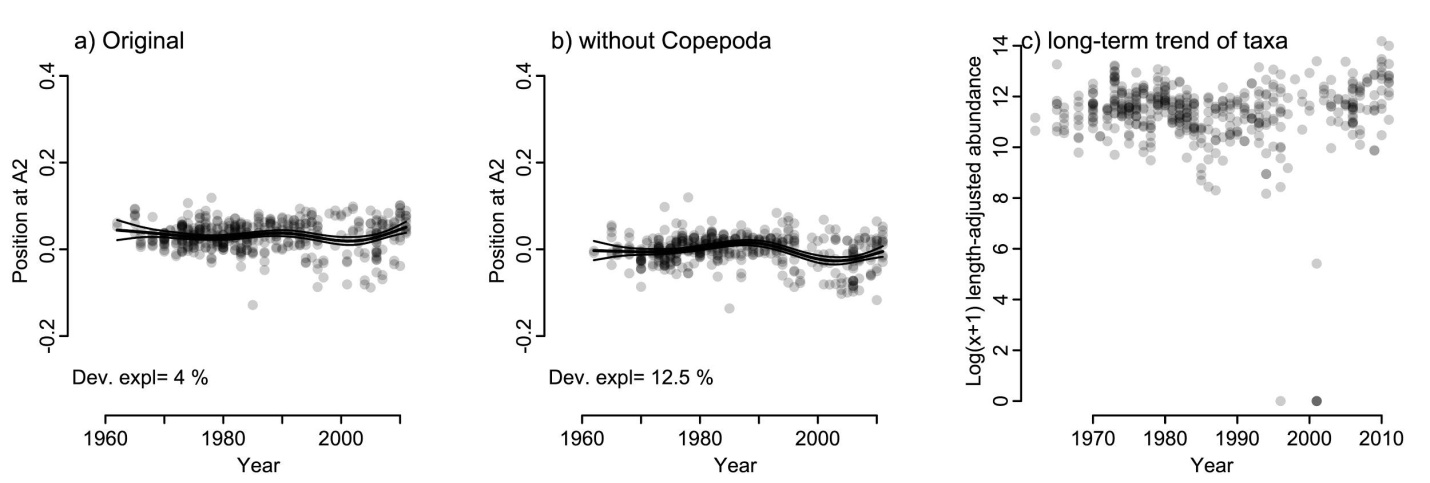


Figure S1f. Coastal, August; position along PCoA axis 2.


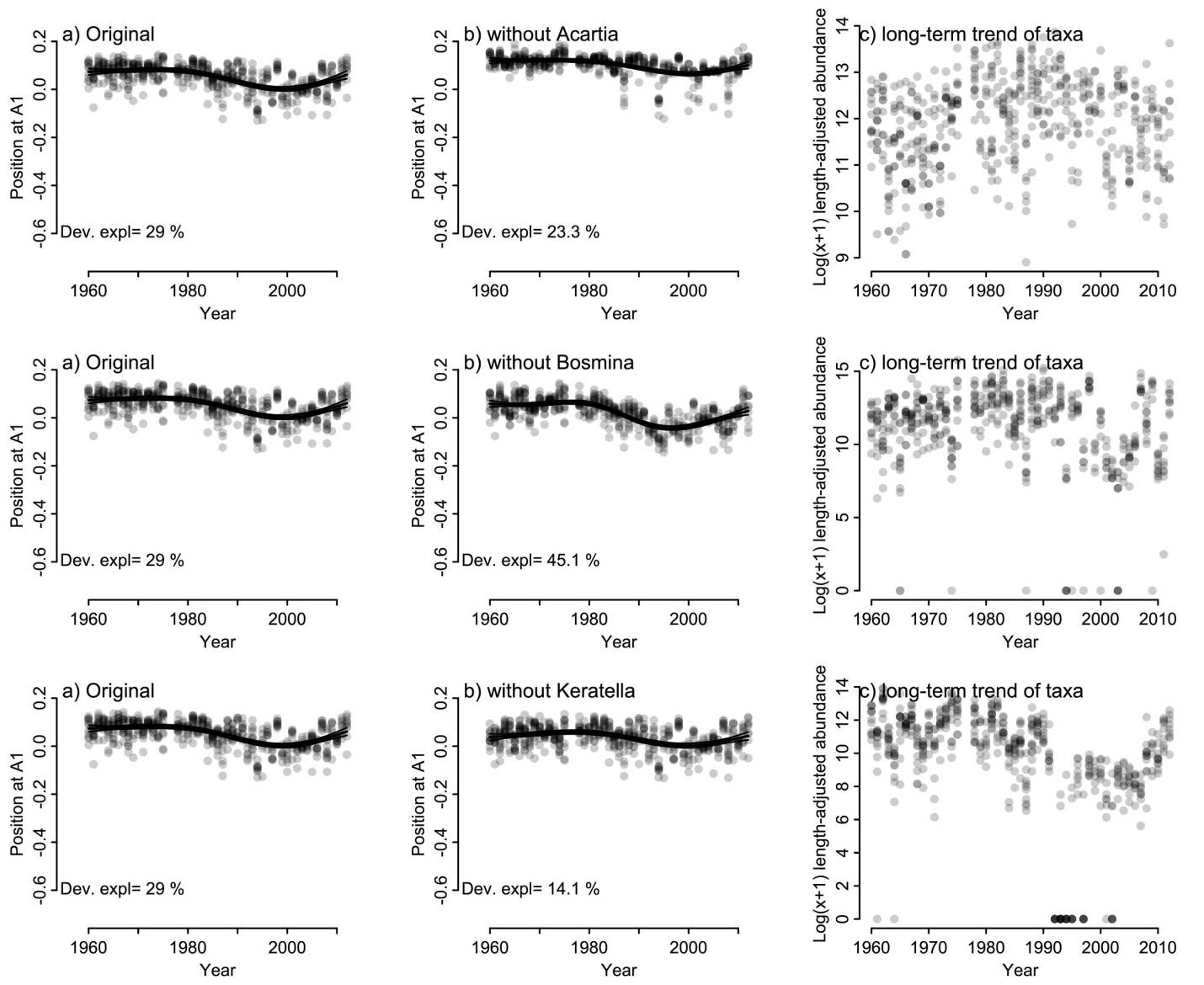


Figure S1g. Open, August; position along PCoA axis 1.


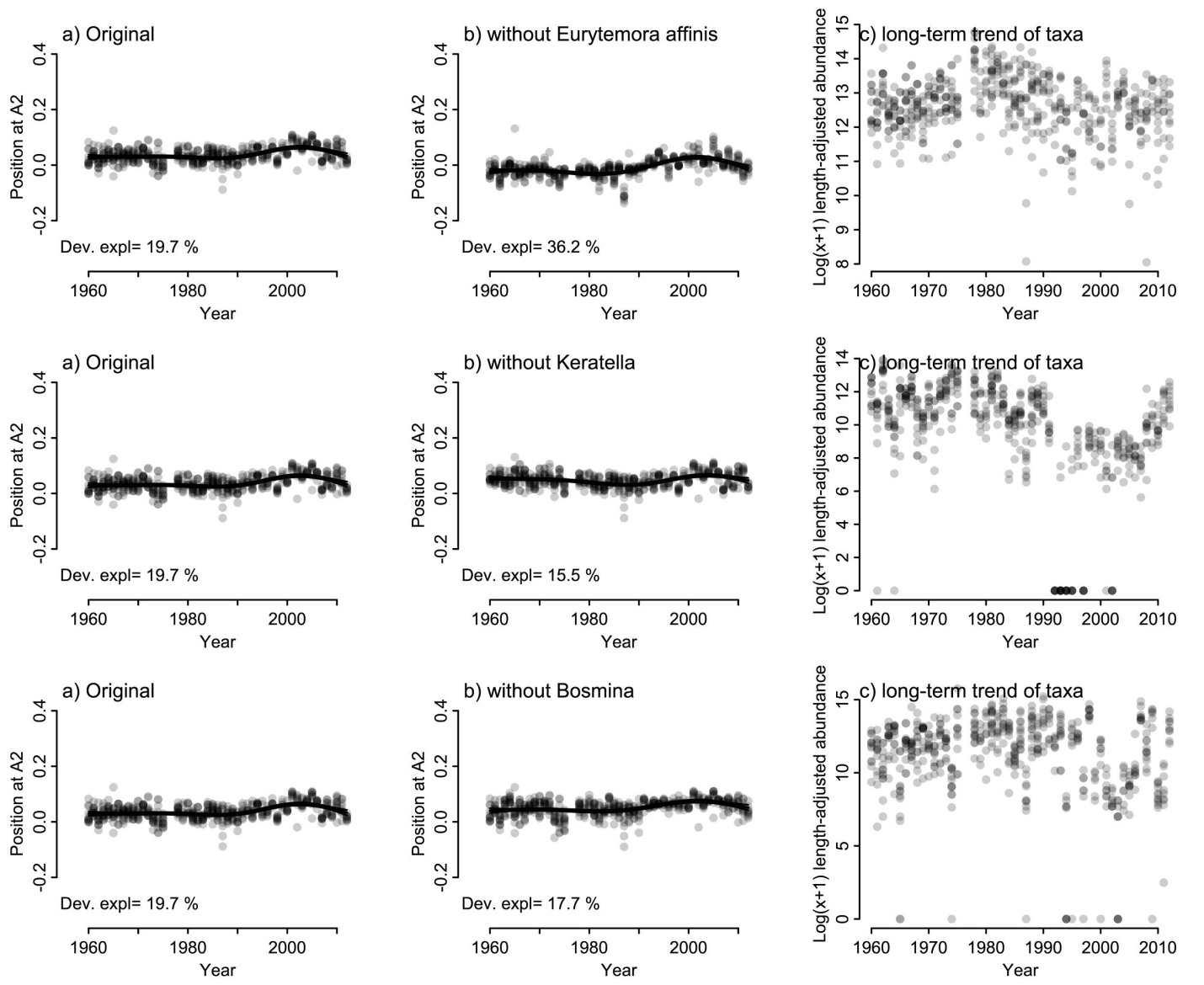


Figure S1h. Open, August; position along PCoA axis 2.
